# Knowledge on Management of Traumatic Dental Injury and Related Predictors in Taif, Saudi Arabia: A Cross-Sectional Study

**DOI:** 10.1155/ijod/3623119

**Published:** 2025-02-07

**Authors:** Bandar S. Shukr, Haifaa M. Alshamrani, Mohammed A. Alzubaidi, Ali A. Alqarni, Muaath H. Alzahrani, Faisal K. Altalhi, Abdulrahman A. Alrizqi

**Affiliations:** ^1^Community Dentistry, Department of Preventive Dentistry, Faculty of Dentistry, Taif University, P.O. Box 11099, Taif 21944, Saudi Arabia; ^2^Faculty of Dentistry, King Abdulaziz University, Jeddah, Saudi Arabia; ^3^Paediatric Dentistry, Department of Preventive Dentistry, Faculty of Dentistry, Taif University, P.O. 11099, Taif 21944, Saudi Arabia; ^4^Immunology and Oral Medicine, Department of Oral and Maxillofacial Surgery and Diagnostic Sciences, Faculty of Dentistry, Taif University, P.O. Box 11099, Taif 21944, Saudi Arabia; ^5^Faculty of Dentistry, Taif University, P.O. Box 11099, Taif 21944, Saudi Arabia; ^6^Restorative Dentistry, Ministry of Health, Taif, Saudi Arabia

**Keywords:** children, dental emergency, dental trauma, factors, knowledge, traumatic dental injury

## Abstract

**Objectives:** This study was undertaken to investigate the knowledge and attitudes toward pediatric traumatic dental injury (TDI) among the population of Taif, as well as predictors of emergency management and intentions for future education, with an additional focus on the subpopulation of parents.

**Methods:** Data were collected from 345 participants using an anonymous questionnaire that was distributed online and contained four sections: demographics, questions regarding TDI knowledge and previous experiences, questions about the management of two clinical cases, and questions about the self-reported capability to manage future TDIs and the motivation for future education/training. Adjusted linear and logistic regressions were utilized to assess the effect of the different predictors on the total knowledge score, self-reported management capabilities, TDI learning importance, and future education/training interests.

**Results:** The findings revealed poor overall knowledge regarding TDI management, especially in avulsion incidents, with slightly better knowledge in the subpopulation of parents. In the total population, working in a governmental job (*p*-value = 0.047) or as a freelancer (*p*-value = 0.006) was associated with higher knowledge, while obtaining any previous TDI information was associated with sufficient management capabilities (*p*-value = 0.001). Regarding the parent subpopulation, attending a previous TDI educational course was associated with higher knowledge (*p*-value = 0.043). Similar to the total population, obtaining any previous TDI information was associated with sufficient management capabilities (*p*-value = 0.001). However, receiving such information from professional resources was associated with lower management capabilities (*p*-value = 0.033). No significant associations were observed regarding the outcomes of TDI learning importance and future education/training interests.

**Conclusion:** This study highlights significant gaps in the knowledge, awareness, and skills related to pediatric dental injuries and their management among the population of the Taif region. Targeted educational interventions are needed to increase public awareness and bridge the current gaps in knowledge and skills.

## 1. Introduction

Traumatic dental injuries (TDIs) that occur to the primary and permanent teeth, as well as the supporting structures, are one of the most prevalent dental issues observed in children [1, 2]. Moreover, over 1 billion individuals globally are impacted by TDIs, and almost 18% of facial injuries were found to be related to TDIs [3]. Unlike many illnesses that can be prevented, TDIs are not always preventable, and anyone can suffer from them at any time [4]. In addition, some studies showed a higher burden for TDIs in children compared to dental caries and periodontal diseases [5, 6]. Globally, the prevalence of TDIs was reported to range between 6% and ~60% [7, 8]. TDI prevalence was also found to be high in a recent systematic review that investigated different healthcare settings, with estimates ranging from 1.88% up to almost 87% [9]. The prevalence rates of TDIs in Saudi Arabia ranged between 31% and 34%, with the upper central incisors being the most impacted teeth, followed by the lower central incisors [10, 11].

Children often sustain accidents during their regular activities, such as riding bicycles, playing soccer, and running, and these accidents might cause dental trauma [12]. A meta-analysis study revealed that the two most frequent places where TDIs occur were schools and homes [12]. In addition, ~60% of a child's time is likely to be spent at home rather than at school or at the playground [13]. Therefore, the parents of the child are most likely to be the first to arrive at the injury site and make emergency decisions, which may directly affect the prognosis of the injured teeth [14, 15]. Even when an injury takes place in a school setting, the school administration is responsible of notifying the parents about any accidental injuries [14]. Hence, to correctly handle such injuries, the parent or the responsible caregiver needs good comprehension, quick application of judgment, and immediate action [14]. Moreover, restoring the dental and emotional health of the injured child is largely dependent on the action of both the parent and caregiver (via their quick response), as well as the treating dentist [14].

The current evidence from both international and local Saudi investigations shows poor levels of understanding regarding TDIs and how to manage them, especially among the parents, with severely insufficient knowledge regarding avulsions to permanent teeth, which require immediate adequate management to avoid any complications in the future, such as inflammatory resorption [16–18]. With the high global prevalence of TDIs in children due to their active lifestyles and the serious long-term consequences sustained from these injuries (e.g., poor self-esteem and quality of life), scientific studies on TDIs are important to help in identifying gaps in awareness and education and improving the ability of the general public, particularly parents and healthcare professionals, to prevent and effectively respond to TDIs in children. In addition, such studies can encourage policymakers and health organizations to develop efficient and targeted intervention strategies, which ultimately contribute to improving the immediate and long-term health outcomes for children worldwide.

The literature shows a dearth of information regarding parents' awareness of TDIs in Saudi Arabia, with most studies conducted on populations other than parents, such as schoolteachers [19], emergency room staff [20], dental students [21], and dentists [22]. This study seeks to fill this gap by focusing on the knowledge and attitudes of the general population in Taif, Saudi Arabia, with a particular emphasis on parents. By identifying predictors of emergency management capabilities and intentions for further education, this research provides critical insights into the local context of TDIs. Unlike previous studies, it uniquely investigates parental awareness and readiness to act in emergencies, offering evidence to inform targeted interventions and policies. By contributing localized data and highlighting the specific needs of parents in Taif, this study adds a crucial dimension to the understanding of TDIs in Saudi Arabia, bridging existing gaps in the literature and paving the way for improved public health strategies.

## 2. Materials and Methods

### 2.1. Study Design and Ethical Considerations

The current study is a cross-sectional, descriptive, survey-based study that used an electronic self-administered structured survey to evaluate the knowledge and attitude of TDIs in children among individuals living in Taif, Saudi Arabia, with an additional focus on those who have children. The study was carried out in Taif city, Saudi Arabia, between November 2023 and June 2024. A consent statement was provided in the first section of the study survey, and participants were deemed to have given their consent to participate if they answered and completed the survey. The Preferred Reporting items for Observational studies in Endodontics (PROBE) 2023 guidelines were followed at the time of manuscript preparation [23].

### 2.2. Study Sample

The inclusion criteria included subjects who were 18 years of age or older, understood the Arabic language, and were residents of Taif city, Saudi Arabia. An additional subgroup analysis was also planned to investigate those with children in the same population. According to the 2022 Saudi Census report [24], the total population of Taif was estimated to be 913,374 persons. Sample size calculation was done using the Raoasft website, with a 95% confidence interval (CI) and 5% marginal error, and it was estimated to be 384 individuals. The study sample consisted of 345 individuals who were conveniently selected, with the exclusion of 20 participants, as they were not residents of Taif city at the time of data collection.

### 2.3. Data Collection Instrument (Study Questionnaire)

Data have been conveniently collected using an anonymous, self-administered, four-part structured online questionnaire. The questionnaire was designed using Google Forms and was distributed via different social media platforms. The study questionnaire comprised of 24 validated, close-ended, multiple-choice questions that were adapted from the study by Al-Sehaibany et al. [14]. In addition, the questions were modified based on a thorough review of previously conducted studies in the current literature, as well as using the most recent dental trauma guidelines published by the International Association of Dental Traumatology (IADT) [25–29]. It was written in English first and then translated into Arabic language. To reduce bias, the questionnaire was pilot-tested for validation in 10 different participants before data collection, and necessary corrections were made, with the responses of those participants excluded from the final study sample. The test group included an equal number of males and females, four of which were parents, with age ranging between 20 and 52 years. Feedback was collected from the pilot participants on their experience and any difficulties they encountered. Based on this, some questions were reformatted to make them clear, understandable, and interpreted consistently by different respondents.

**Figure 1:**
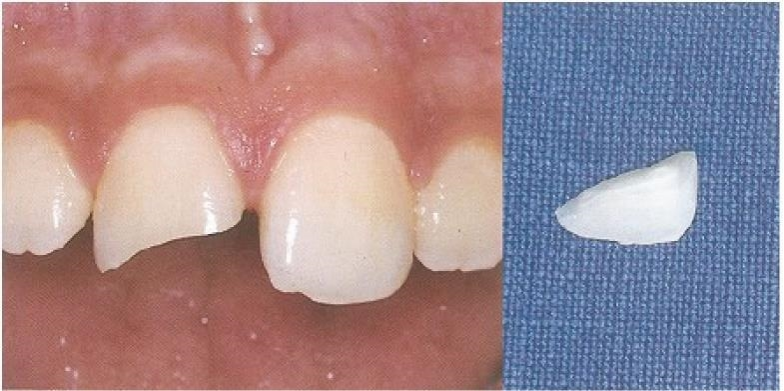
Case−1: An 11-year-old child fell and broke an upper front tooth.

**Figure 2:**
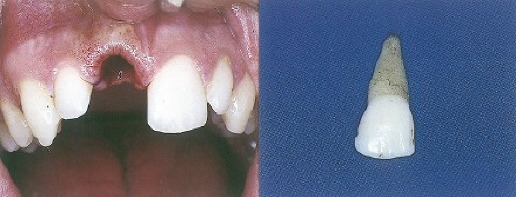
Case−2: A 9-year-old child fell, and the upper front tooth got knocked out.

The questionnaire was divided into four parts in which each part was intended to gather different information. To reduce information bias expected in self-administered survey studies, an overview of the study and its objectives, with highlights on the importance of providing accurate answers, was included at the beginning of the questionnaire. Additionally, a consent statement ensuring confidentiality of the collected data and the right to withdraw at any time was also included. The first part included questions about demographic data, including age, gender, marital status, nationality, level of education, occupation, number of children, if any, and whether the participant was living in the city of Taif or not at the time of survey administration. The second part contained six questions regarding TDI knowledge and previous experiences. The third part was about the proper management of two clinical case scenarios, with clinical pictures; both were adapted from the textbook *Essentials of Traumatic Injuries to the Teeth: A Step-by-Step Treatment Guide* [30]. An 11-year-old child who had fallen and fractured his upper front teeth was the subject of the first scenario (permanent tooth fracture) (Figure 1). The second scenario was about a 9-year-old child who had fallen and knocked out his upper front tooth (permanent tooth avulsion) (Figure 2). For each scenario, different questions were provided to the respondents, with one correct answer for each question. A total of seven questions were included in this part, with one point given for each correct answer, and the sum of the scores for all the correct answers was used to determine the total knowledge score. The maximum possible knowledge score was 7, and the lowest score was zero, in case a respondent provided incorrect answers in all seven questions. The final part was about the subjective self-reported capabilities of the respondents to manage TDIs if they happen in the future and the motivation for obtaining further education and training in TDIs.

### 2.4. Statistical Analysis

Frequencies and percentages were computed to describe the data as appropriate. The effect of the different demographic factors on the total knowledge score, as well as the effect of factors related to previous TDI experiences, was assessed using adjusted multiple linear regression models. In addition, adjusted logistic regression models were used to explore the effect of the different predictors on participants' self-reported TDI management capabilities, TDI learning importance, and future interest in education and training. The results were presented as 95% CIs, adjusted beta coefficients for linear models, and adjusted odds ratios (AORs) for logistic models, respectively. An additional subgroup analysis was conducted to only investigate the parents enrolled in the study. The residual plots were used to confirm the homogeneity of variances, while the Kolmogorov–Smirnov test and histogram plots were used to evaluate the data's normality. The variance inflation factor (VIF) was used to detect any problems related to multicollinearity across the study variables. In addition, Cook's distance was used to identify significant outliers. For the models' goodness of fit, the adjusted coefficient of determination (Adj. *R*^2^) was utilized in the linear regression, while the Hosmer and Lemeshow test was used in the logistic analysis.

In all models, no abnormalities were detected regarding the different assumptions (i.e., data normality, VIF,…). However, a multicollinearity issue (VIF > 10) was detected between the variables of “marital status” and “number of children” in all analyses; therefore, “marital status” was removed from all the models. Similarly, the same issue was identified between “education” and “occupation” in the analysis of the parent subpopulation, and as a result, “occupation” was removed from all the models in that analysis. In addition, estimates for some categories could not be generated due to the small number of observations in these categories; hence, collapsing was performed when possible. All statistical procedures were two-tailed, with a significance level of 0.05 or less. All the collected data from the Google Forms link were exported as an Excel spreadsheet and were analyzed via SPSS software (SPSS version 25.0; Chicago, IL, USA).

## 3. Results

The study sample consisted of 345 participants, with a majority being females (87.1%). Most of the participants (47.4%) were 18–24 years of age. Regarding educational attainment, 44.6% had a bachelor's degree or higher, followed by 38.8% who had completed high school. Occupationally, the largest group was students (45.7%), followed by government employees (22.8%). Marital status showed that 49.1% were married, and 47.6% were single. Most participants were Saudi nationals (96.3%).

In terms of family dynamics, 47.7% had children, with 35.7% having three or more children. A significant portion (55.1%) had witnessed a recent TDI accident, with 26.2% of these involving their own children. Only 13.2% had attended a TDI educational course, and 49.5% had obtained TDI information, primarily from dentists (25.8%) and the internet/social media (12.9%) (Table 1).

**Figure 3:**
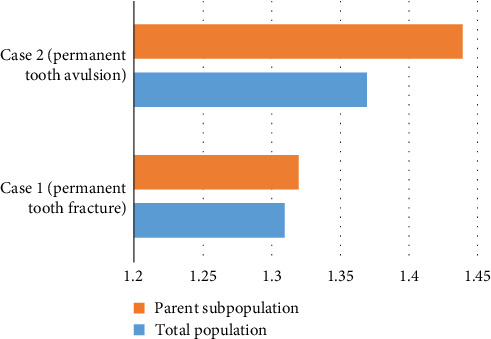
Average knowledge of the study participants regarding traumatic dental injury (TDI) management in two clinical case scenarios.

The analytical sample included 325 participants, with a subset of 155 parents with children. Knowledge regarding TDI management was assessed through two clinical case scenarios. In the first case (permanent tooth fracture), less than half of the participants in both the total population (41.2%) and the parent subpopulation (43.2%) correctly identified that the broken piece should be saved. Most of the participants in both groups (90.2% of the total population and 88.4% of parents) correctly indicated the immediate action that should be taken in this situation. Regarding the second clinical case (permanent tooth avulsion), 32.3% of the total population and 36.1% of parents correctly identified the type of knocked-out tooth. However, only very few (11.7% of the total population, 12.3% of parents) correctly knew the immediate action that should be taken in this scenario. Similarly, only a small group (around 24% of both the total population and the parents) correctly indicated the part that should be touched to carry the knocked-out tooth. A similar observation was noticed regarding the best storage medium (around 28% correct answers in both groups). Finally, less than half of the participants in both groups (around 40%) correctly identified the time frame for the tooth replantation to be successful (Table 2).  Figure 3 illustrates the average knowledge of participants in the two clinical case scenarios. The findings indicate that parents had slightly higher average knowledge scores in both scenarios compared to the total population.

The association between the different predictors and the total knowledge score regarding TDI management was also assessed in this study (Table 3). In the total population group, significant associations were observed between the total knowledge score and occupation, witnessing a recent TDI accident, and attending a TDI educational course in the past. Specifically, participants who were working in a government job had a 1.08 higher average knowledge score compared to those who were students (*p*-value = 0.047). Likewise, those who were freelancers had higher TDI knowledge by 1.98 points (*p*-value = 0.006). In addition, participants who witnessed a recent TDI accident had 0.35 higher TDI knowledge, while those who attended a TDI educational course had 0.50 higher knowledge; however, the effects were borderline significant (*p*-value = 0.098 and *p*-value = 0.074, respectively). In the subpopulation of parents, only those who attended a TDI educational course had a significantly higher TDI knowledge by 0.86 points compared to those who did not attend any courses (*p*-value = 0.043).

The associations between the different predictors and outcomes related to the self-reported capability to manage future TDI incidents, perceived TDI learning importance, and the interest to receive future TDI education/training were also analyzed in the present investigation. In the total population group, participants who obtained any TDI information in the past were more likely to report sufficient capabilities to correctly manage any future TDI accidents compared to those who did not obtain any previous information (AOR = 4.77, *p*-value = 0.001). A similar effect was also observed among male participants compared to the female ones (AOR = 2.09). On the other hand, participants of non-Saudi nationality were less likely to report sufficient TDI management capabilities (AOR = 0.027). However, the effect of sex and nationality were found to be borderline significant (*p*-value for sex = 0.085, *p*-value for nationality = 0.070). No significant associations were found among the group regarding the outcomes of perceived TDI learning importance and the interest to receive future TDI education/training (Table 4).

In the parent group, and similar to the total population group, parents who obtained any TDI information in the past were more likely to report sufficient capabilities to accurately manage any future TDI incidents compared to those who did not obtain any previous information (AOR = 7.65, *p*-value = 0.001). Surprisingly, parents who received this information from a professional source (i.e., a dentist or physician) were less likely to report sufficient TDI management capabilities (AOR = 0.29, *p*-value = 0.033). Additionally, parents who attended any previous TDI educational course were more likely to be interested in receiving future TDI education/training compared to those who did not attend any TDI course in the past (AOR = 7.73). However, the association was of a borderline significance (*p*-value = 0.070). Moreover, no significant association was identified among the group regarding the outcome of perceived TDI learning importance (Table 5).

## 4. Discussion

TDIs in children are a significant public health concern, with many incidents have been documented around the world. Such incidents can have serious health consequences for the affected child, especially if early and proper management was not received. Research on TDIs is crucial to help in understanding their prevalence and related factors, improving the treatment outcomes and the quality of life for the affected children, as well as promoting effective healthcare policies and practices. The present study is significant as it investigates the knowledge and attitudes toward pediatric TDIs, as well as the factors related to the management capabilities and intentions for future education among the population of Taif, with an emphasis on parents who have children. The findings revealed insufficient knowledge of proper TDI management among participants, particularly in handling tooth avulsions. Although parents demonstrated slightly higher knowledge levels compared to the general population, many were unaware of critical steps, such as timely replantation or appropriate storage media for avulsed teeth.

The results of this study showed a lack of general understanding about pediatric TDIs and the appropriate management practices among the sampled individuals, especially in cases of accidents involving permanent teeth avulsions, with slightly better knowledge noticed among parents who had children. These results align with prior studies in Saudi Arabia and globally. A recent systematic review reported insufficient levels of parental knowledge, awareness, and skills to appropriately manage pediatric dental trauma in most of the included studies, which could result in poor long-term prognosis in the traumatized teeth [31]. Additionally, the total TDI knowledge and management scores were found to be “below satisfactory levels” in another recent investigation conducted by Momeni, Afzalsoltani, and Moslemzadehasl [32] on a sample of mothers. In Saudi Arabia, a similar pattern of poor TDI management knowledge was also reported in a study conducted on a group of mothers in the region of Al-Qassim [17], as well as in another study that included parents in the same region [33]. Lack of adequate knowledge among the parents was also reported in a study that was conducted in two heavily populated locations in Saudi Arabia, the Eastern Province and the city of Riyadh [34]. These studies collectively highlight the significant implication of improving parental awareness of TDI management, particularly for tooth avulsions, through comprehensive public health campaigns and school-based programs that are focused on basic first aid training for dental trauma.

The lack of hands-on training and practical knowledge among the general population is particularly concerning, given the high prevalence of TDIs among children, which often demand immediate and skilled intervention to prevent long-term complications. Examples of interventions that could be beneficial include providing regular community-based training sessions on TDIs and the incorporation of basic first aid training courses for dental trauma into high school and undergraduate university curriculums, as well as into public health education programs, to enhance the community's response to such emergencies. Moreover, the use of innovative teaching resources, such as virtual simulations and interactive case studies, may further enhance the learning experience and outcomes.

The relationships between TDI management and the different predictors were also examined in the present study. The findings showed significantly higher knowledge among individuals who were working in a governmental job. The existing evidence does not show a direct link between the type of employment and having greater TDI knowledge. Nevertheless, employment status was found to be significantly associated with higher TDI knowledge, as reported in the study by Momeni, Afzalsoltani, and Moslemzadehasl [32] which showed higher knowledge among mothers who worked compared to those who were unemployed. The high knowledge among governmental employees might be because they usually have better access to learning resources and training programs, such as workshops and seminars, to achieve continuous professional development. These resources help them advance their knowledge and abilities in a variety of fields, which could include TDI management. Additionally, government workers could be more exposed to health and safety information through their work environments, as they often have roles or responsibilities that directly impact the health of the community. The present findings also showed a similar pattern of higher knowledge among participants who were working as freelancers. One possible explanation is the higher networking opportunities. Freelancers may have greater opportunities to network with experts in various professions, including healthcare. Such networking might facilitate the exchange of knowledge and best practices related to TDI management. The role of employment-related factors suggests the potential for workplace-based educational programs to improve awareness in specific demographic groups. Expanding these programs to reach underrepresented populations, such as non-Saudi residents, could also reduce disparities in access to dental trauma care and information.

A higher level of knowledge regarding dental injuries was also found among individuals who attended a TDI educational course in the past, as well as those who obtained any previous information about TDI. This was parallel with the previously conducted study on a group of mothers in the region of Al-Qassim, Saudi Arabia [17]. This was expected as these previous courses and information mostly provided them with structured, updated, and comprehensive knowledge about TDIs. In addition, some of these courses might included practical, hands-on training exercises, where they can practice the skills they learned to manage dental injuries.

One of the interesting findings was that parents who received TDI information from a professional source (i.e., a dentist or physician) were less likely to report sufficient capabilities to manage any future dental injuries. One possible justification is that most of these parents were hesitant or lacked enough confidence to manage future dental injuries. While having sufficient knowledge is critical to manage traumatic injuries, this knowledge can be ineffective when it comes to saving the traumatized tooth if it is not coupled with enough confidence during the management process. Another possible justification for this is the complexity of information. Professionals usually provide dense and complex information that can be sometimes overwhelming. This intricacy may possibly lead to individuals feeling less competent to manage similar situations on their own. In addition, learning about the potential risks and complications associated with dental trauma from a professional healthcare provider might increase the levels of anxiety and reduce self-confidence. Being aware of the worst-case scenarios might make the persons more apprehensive about managing these injuries themselves. Lack of practical experience might also play a role in the TDI self-management capabilities, making the individuals feel less prepared to handle dental injuries on their own without assistance from a specialist. The association between previous educational exposure and higher knowledge levels suggests that structured training sessions or workshops could significantly enhance TDI management capabilities. Furthermore, integrating practical demonstrations and scenario-based training into these programs could help address the confidence deficit observed among parents, especially those who received professional guidance but felt uncertain about applying it. Simplifying professional advice into actionable steps may further empower individuals to act decisively during emergencies.

The findings also have implications for healthcare professionals and policymakers. Dental practitioners, who are often a primary source of TDI information, should consider tailoring their communication strategies to ensure clarity and reduce the anxiety of caregivers. Policymakers could support these efforts by mandating the inclusion of dental trauma management in community health initiatives and by fostering collaborations between schools, healthcare providers, and public health organizations.

While the study highlighted factors such as occupation, previous exposure, and educational courses as predictors of TDI knowledge, some associations, like the impact of gender and nationality, were found to be borderline significant. Collectively, all of these borderline associations worth further investigations, with a sufficient number of subjects in the relevant categories, to better understand TDI and related factors.

Despite the study's strengths, including its focus on parents and use of rigorous statistical analyses, limitations such as potential biases from self-reported data and the cross-sectional design should be acknowledged. Larger, longitudinal studies across Saudi Arabia are needed to generalize findings and develop comprehensive strategies for improving TDI management awareness and skills.

## 5. Conclusions

Overall, the findings of this study clearly demonstrated a substantial deficiency in TDI awareness and management among the general population of the Taif region, with slightly greater knowledge among the subpopulation of parents. In the total population, working in a governmental job or as a freelancer was associated with higher TDI knowledge, while obtaining any previous TDI information was associated with sufficient capabilities to manage future TDI incidents. In the parent group, attending a TDI educational course in the past was associated with higher TDI knowledge. Additionally, like the total population, obtaining any previous TDI information was associated with sufficient TDI management capabilities. These findings underscore the need for holistic approaches that combine education, policy support, and practical interventions to enhance the community's ability to manage TDIs effectively, thereby minimizing long-term complications and improving children's health outcomes.

## Figures and Tables

**Table 1:** Characteristics of the study sample (*N* = 345).

Characteristic	*n* (%)
Sex	
Males Females	42 (12.9) 283 (87.1)
Age (years)	
18–24 years 25–34 years 35–44 years 45 years or older	154 (47.4) 25 (7.7) 76 (23.4) 70 (21.5)
Education	
Primary school Middle school High school Diploma Bachelor's degree or higher No formal education	5 (1.5) 26 (8.0) 126 (38.8) 22 (6.8) 145 (44.6) 1 (0.3)
Occupation	
Governmental Private sector Freelancer Unemployed Retired Student	74 (22.8) 20 (6.2) 7 (2.2) 47 (14.5) 28 (8.6) 149 (45.7)
Marital status	
Single Married Divorced Widowed	154 (47.6) 160 (49.1) 6 (1.8) 5 (1.5)
Nationality	
Saudi Non-Saudi	313 (96.3) 12 (3.7)
Have children	
Yes No	155 (47.7) 170 (52.3)
Number of children	
1 2 3 children or more Do not have any children	19 (5.8) 20 (6.2) 116 (35.7) 170 (52.3)
Witnessed any recent TDI accident in the past	
Yes No	179 (55.1) 146 (44.9)
Relation to the injured child in that accident	
A son or daughter A relative A neighbor A stranger Never witnessed any TDI accident before	85 (26.2) 83 (25.5) 3 (0.9) 8 (2.5) 146 (44.9)
Attended any TDI educational course in the past	
Yes No	43 (13.2) 282 (86.8)
Obtained any TDI information in the past	
Yes No	161 (49.5) 164 (50.5)
If “yes,” source of information	
Dentist Physician Friend or relative Internet/social media Other Never obtained TDI information in the past	84 (25.8) 3 (0.9) 15 (4.6) 42 (12.9) 17 (5.2) 164 (50.6)

Abbreviation: TDI, traumatic dental injury.

**Table 2:** Knowledge of the study participants regarding TDI management in two clinical case scenarios.

No.	Item	Total population (*N* = 325)		Parent subpopulation (*N* = 155)	
		Correct	(%)	Correct	(%)
**Case 1: Permanent tooth fracture**					

1	Should the broken piece of the tooth be saved? - Yes - No - I do not know	134	41.2	67	43.2
2	Immediate action should be: - Send the child immediately to the dentist - Put the child to bed to sleep - I do not know	293	90.2	137	88.4

**Case 2: Permanent tooth avulsion**					

3	The knocked-out tooth is likely to be: - Baby tooth - Permanent tooth - I do not know	105	32.3	56	36.1
4	What will be your immediate action? - Replant the tooth and send the child to the dentist - Save it in a storage medium and send the child to the dentist - Stop the bleeding and have the child rest - Not sure what to do	38	11.7	19	12.3
5	The knocked-out tooth should be carried by: - The crown - The root - Any part - I do not know	79	24.3	38	24.5
6	In case the knocked-out tooth needs to be stored in a storage medium, which of the following is the best storage medium? - Tap water - Antiseptic solution - Tissue paper - Milk or the child's own saliva - Normal saline - I do not know	91	28.0	46	29.7
7	How soon should the knocked-out tooth be replanted? - Immediately - Within 30 min - Within a few hours - By the next day - No need to visit a dentist - I do not know	133	40.9	64	41.3

Abbreviation: TDI, traumatic dental injury.

**Table 3:** Associations between the different predictors and the total knowledge score regarding TDI management.

Predictor	Total population (*N* = 325)		Parent subpopulation (*N* = 155)	
	*β* coefficient (95% CI)	*p*-Value	*β* coefficient (95% CI)	*p*-Value
Sex (female)	Reference	Reference	Reference	Reference
Sex (male)	−0.14 (−0.73–0.43)	0.619	−0.07 (−0.68–0.53)	0.798
Age (18–24 years)	Reference	Reference	Reference	Reference
Age (25–34 years)	−0.21 (−1.15–0.71)	0.644	−0.68 (−1.95–0.59)	0.293
Age (35–44 years)	−0.48 (−1.44–0.47)	0.324	−0.43 (−1.51–0.63)	0.420
Age (45 years or older)	−0.07 (−1.09–0.94)	0.885	−0.01 (−1.10–1.08)	0.984
Education (high school or less)	Reference	Reference	Reference	Reference
Education (more than high school)	−0.20 (−0.67–0.25)	0.381	0.10 (−0.50 – 0.70)	0.745
Occupation (student)	Reference	Reference	—	—
Occupation (governmental)	1.08 (0.01–2.14)	0.047*⁣*^*∗*^	—	—
Occupation (private sector)	0.60 (−0.47–1.68)	0.270	—	—
Occupation (freelancer)	1.98 (0.56–3.40)	0.006*⁣*^*∗*^	—	—
Occupation (retired)	0.31 (−0.88–1.50)	0.610	—	—
Occupation (unemployed)	0.69 (−0.28–1.66)	0.163	—	—
Nationality (Saudi)	Reference	Reference	Reference	Reference
Nationality (non-Saudi)	−0.02 (−0.93–0.89)	0.965	0.31 (−0.82–1.45)	0.581
Number of children (do not have any children)	Reference	Reference	—	—
Number of children (less than 3 children)	−0.09 (−0.82–0.63)	0.792	Reference	Reference
Number of children (3 children or more)	−0.15 (−0.80–0.50)	0.651	−0.19 (−0.82–0.45)	0.558
Witnessed any recent TDI accident in the past (no)	Reference	Reference	Reference	Reference
Witnessed any recent TDI accident in the past (yes)	0.35 (−0.06–0.72)	0.098	*⁣* ^*∗∗*^	*⁣* ^*∗∗*^
Relation to the injured child in that accident (never witnessed any TDI accident before)	Reference	Reference	Reference	Reference
Relation to the injured child in that accident (a son or daughter)	−0.34 (−0.88–0.19)	0.209	*⁣* ^*∗∗*^	*⁣* ^*∗∗*^
Relation to the injured child in that accident (a relative/neighbor/stranger)	*⁣* ^*∗∗*^	*⁣* ^*∗∗*^	*⁣* ^*∗∗*^	*⁣* ^*∗∗*^
Attended any TDI educational course in the past (no)	Reference	Reference	Reference	Reference
Attended any TDI educational course in the past (yes)	0.50 (−0.04–1.06)	0.074	0.86 (0.02–1.69)	0.043*⁣*^*∗*^
Obtained any TDI information in the past (no)	Reference	Reference	Reference	Reference
If “yes,” source of information (never obtained TDI information in the past)	Reference	Reference	Reference	Reference
If “yes,” source of information (dentist or physician)	−0.03 (−0.46–0.39)	0.887	0.08 (−0.53 – 0.70)	0.777
If “yes,” source of information (friend or relative/internet/social media/other)	0.20 (−0.26–0.67)	0.388	0.52 (−0.12 – 1.17)	0.114

*Note:* Dependent variable: total knowledge score regarding TDI management.

Abbreviations: CI, confidence interval; TDI, traumatic dental injury.

*⁣* ^*∗*^ *p*-Value ≤ 0.05 was considered statistically significant.

*⁣* ^*∗∗*^The parameter could not be estimated because of the small number of subjects in this category.

**Table 4:** Associations between the different predictors and TDI management, perceived TDI learning importance, and future education/training among the total population (*N* = 325).

Predictor	TDI management ◆		TDI learning importance ¶		TDI future education/training ɸ	
	AOR (95% CI)	*p*-Value	AOR (95% CI)	*p*-Value	AOR (95% CI)	*p*-Value
Sex (female)	Reference	Reference	Reference	Reference	Reference	Reference
Sex (male)	2.09 (0.90–4.84)	0.085	*⁣* ^*∗∗*^	*⁣* ^*∗∗*^	0.49 (0.18–1.33)	0.167
Age (18–24 years)	Reference	Reference	Reference	Reference	Reference	Reference
Age (25–34 years)	0.56 (0.13–2.35)	0.431	0.17 (0.01–3.58)	0.258	0.43 (0.07–2.49)	0.353
Age (35–44 years)	0.69 (0.16–2.92)	0.623	*⁣* ^*∗∗*^	*⁣* ^*∗∗*^	0.24 (0.03–1.78)	0.165
Age (45 years or older)	0.69 (0.14–3.04)	0.603	*⁣* ^*∗∗*^	*⁣* ^*∗∗*^	0.47 (0.05–3.92)	0.489
Education (high school or less)	Reference	Reference	Reference	Reference	Reference	Reference
Education (more than high school)	0.98 (0.50–1.91)	0.965	0.70 (0.09–5.32)	0.731	1.29 (0.59–2.81)	0.521
Occupation (student)	Reference	Reference	Reference	Reference	Reference	Reference
Occupation (governmental)	0.57 (0.11–2.85)	0.501	*⁣* ^*∗∗*^	*⁣* ^*∗∗*^	4.42 (0.52–37.69)	0.173
Occupation (private sector)	1.60 (0.32–7.84)	0.561	*⁣* ^*∗∗*^	*⁣* ^*∗∗*^	2.86 (0.38–21.36)	0.306
Occupation (freelancer)	5.66 (0.46–68.83)	0.174	*⁣* ^*∗∗*^	*⁣* ^*∗∗*^	*⁣* ^*∗∗*^	*⁣* ^*∗∗*^
Occupation (retired)	0.33 (0.05–2.14)	0.248	*⁣* ^*∗∗*^	*⁣* ^*∗∗*^	1.15 (0.12–11.00)	0.904
Occupation (unemployed)	1.44 (0.34–6.16)	0.617	*⁣* ^*∗∗*^	*⁣* ^*∗∗*^	2.59 (0.36–18.66)	0.343
Nationality (Saudi)	Reference	Reference	Reference	Reference	Reference	Reference
Nationality (non-Saudi)	0.27 (0.06–1.11)	0.070	*⁣* ^*∗∗*^	*⁣* ^*∗∗*^	3.26 (0.39–27.25)	0.274
Number of children (do not have any children)	Reference	Reference	Reference	Reference	Reference	Reference
Number of children (less than 3 children)	1.80 (0.61–5.32)	0.284	0.14 (0.01–2.76)	0.198	1.13 (0.31–4.03)	0.844
Number of children (3 children or more)	1.99 (0.74–5.37)	0.172	0.11 (0.01–1.81)	0.124	1.35 (0.46–3.99)	0.580
Witnessed any recent TDI accident in the past (no)	Reference	Reference	Reference	Reference	Reference	Reference
Witnessed any recent TDI accident in the past (yes)	0.97 (0.53–1.78)	0.929	1.20 (0.23–6.29)	0.827	1.17 (0.59–2.31)	0.649
Relation to the injured child in that accident (never witnessed any TDI accident before)	Reference	Reference	Reference	Reference	Reference	Reference
Relation to the injured child in that accident (a son or daughter)	1.57 (0.71–3.45)	0.256	0.30 (0.01–5.89)	0.430	1.30 (0.50–3.36)	0.578
Relation to the injured child in that accident (a relative/neighbor/stranger)	*⁣* ^*∗∗*^	*⁣* ^*∗∗*^	*⁣* ^*∗∗*^	*⁣* ^*∗∗*^	*⁣* ^*∗∗*^	*⁣* ^*∗∗*^
Attended any TDI educational course in the past (no)	Reference	Reference	Reference	Reference	Reference	Reference
Attended any TDI educational course in the past (yes)	1.08 (0.49–2.35)	0.841	*⁣* ^*∗∗*^	*⁣* ^*∗∗*^	1.13 (0.45–2.85)	0.786
Obtained any TDI information in the past (no)	Reference	Reference	Reference	Reference	Reference	Reference
Obtained any TDI information in the past (yes)	4.77 (2.42–9.40)	0.001*⁣*^*∗*^	*⁣* ^*∗∗*^	*⁣* ^*∗∗*^	0.68 (0.31–1.46)	0.326
If “yes,” source of information (never obtained TDI information in the past)	Reference	Reference	Reference	Reference	Reference	Reference
If “yes,” source of information (dentist or physician)	0.61 (0.31–1.21)	0.162	*⁣* ^*∗∗*^	*⁣* ^*∗∗*^	0.93 (0.43–2.04)	0.873
If “yes,” source of information (friend or relative/internet/social media/other)	*⁣* ^*∗∗*^	*⁣* ^*∗∗*^	*⁣* ^*∗∗*^	*⁣* ^*∗∗*^	*⁣* ^*∗∗*^	*⁣* ^*∗∗*^

Abbreviations: AOR, adjusted odds ratio; CI, confidence interval; TDI, traumatic dental injury.

◆Dependent variable: self-reported capability to manage TDI if happens in the future (yes vs. no).

¶Dependent variable: perceived importance regarding TDI learning (very important/important vs. not important).

**ɸ**Dependent variable: interest to received TDI education/training in the future (yes vs. no).

*⁣* ^*∗*^ *p*-Value ≤ 0.05 was considered statistically significant.

*⁣* ^*∗∗*^The parameter could not be estimated because of small number of subjects in this category.

**Table 5:** Associations between the different predictors and TDI management, perceived TDI learning importance, and future education/training among the subpopulation of parents (*N* = 155).

Predictor	TDI management ◆		TDI learning importance ¶		TDI future education/training ɸ	
	AOR (95% CI)	*p*-Value	AOR (95% CI)	*p*-Value	AOR (95% CI)	*p*-Value
Sex (female)	Reference	Reference	Reference	Reference	Reference	Reference
Sex (male)	—	—	0.23 (0.01–5.51)	0.369	0.42 (0.15–1.18)	0.104
Age (18–24 years)	Reference	Reference	Reference	Reference	Reference	Reference
Age (25–34 years)	2.91 (0.41–20.65)	0.283	*⁣* ^*∗∗*^	*⁣* ^*∗∗*^	0.57 (0.40–8.30)	0.685
Age (35–44 years)	3.19 (0.60–16.94)	0.172	*⁣* ^*∗∗*^	*⁣* ^*∗∗*^	0.73 (0.06–7.72)	0.795
Age (45 years or older)	1.70 (0.31–9.21)	0.536	*⁣* ^*∗∗*^	*⁣* ^*∗∗*^	1.03 (0.09–11.32)	0.977
Education (high school or less)	Reference	Reference	Reference	Reference	Reference	Reference
Education (more than high school)	0.54 (0.22–1.31)	0.177	*⁣* ^*∗∗*^	*⁣* ^*∗∗*^	0.49 (0.15–1.60)	0.241
Nationality (Saudi)	Reference	Reference	Reference	Reference	Reference	Reference
Nationality (non-Saudi)	0.60 (0.12–2.98)	0.536	*⁣* ^*∗∗*^	*⁣* ^*∗∗*^	*⁣* ^*∗∗*^	*⁣* ^*∗∗*^
Number of children (less than 3 children)	Reference	Reference	Reference	Reference	Reference	Reference
Number of children (3 children or more)	0.83 (0.32–2.13)	0.699	0.62 (0.02–16.20)	0.776	0.64 (0.18–2.21)	0.484
Witnessed any recent TDI accident in the past (no)	Reference	Reference	Reference	Reference	Reference	Reference
Witnessed any recent TDI accident in the past (yes)	0.54 (0.17–1.74)	0.305	0.38 (0.01–10.70)	0.572	0.52 (0.13–1.98)	0.343
Relation to the injured child in that accident (never witnessed any TDI accident before)	Reference	Reference	Reference	Reference	Reference	Reference
Relation to the injured child in that accident (a son or daughter)	2.12 (0.69–6.52)	0.187	2.71 (0.09–77.48)	0.559	2.15 (0.61–7.53)	0.229
Relation to the injured child in that accident (a relative/neighbor/stranger)	*⁣* ^*∗∗*^	*⁣* ^*∗∗*^	*⁣* ^*∗∗*^	*⁣* ^*∗∗*^	*⁣* ^*∗∗*^	*⁣* ^*∗∗*^
Attended any TDI educational course in the past (no)	Reference	Reference	Reference	Reference	Reference	Reference
Attended any TDI educational course in the past (yes)	2.54 (0.73–8.86)	0.143	*⁣* ^*∗∗*^	*⁣* ^*∗∗*^	7.73 (0.84–70.43)	0.070
Obtained any TDI information in the past (no)	Reference	Reference	Reference	Reference	Reference	Reference
Obtained any TDI information in the past (yes)	7.65 (2.66–21.95)	0.001*⁣*^*∗*^	*⁣* ^*∗∗*^	*⁣* ^*∗∗*^	0.49 (0.14–1.64)	0.248
If “yes,” source of information (never obtained TDI information in the past)	Reference	Reference	Reference	Reference	Reference	Reference
If “yes,” source of information (dentist or physician)	0.29 (0.09–0.90)	0.033*⁣*^*∗*^	*⁣* ^*∗∗*^	*⁣* ^*∗∗*^	0.87 (0.26–2.93)	0.829
If “yes,” source of information (friend or relative/Internet/social media/other)	*⁣* ^*∗∗*^	*⁣* ^*∗∗*^	*⁣* ^*∗∗*^	*⁣* ^*∗∗*^	*⁣* ^*∗∗*^	*⁣* ^*∗∗*^

Abbreviations: AOR, adjusted odds ratio; CI, confidence interval; TDI, traumatic dental injury.

◆Dependent variable: self-reported capability to manage TDI if happens in the future (yes vs. no).

¶Dependent variable: perceived importance regarding TDI learning (very important/important vs. not important).

**ɸ**Dependent variable: interest to received TDI education/training in the future (yes vs. no).

*⁣* ^*∗*^ *p*-Value ≤ 0.05 was considered statistically significant.

*⁣* ^*∗∗*^The parameter could not be estimated because of the small number of subjects in this category.

## Data Availability

The data that support the findings of this study are available from the upon reasonable request.
